# Cerebral Activation during Von Frey Filament Stimulation in Subjects with Endothelin-1-Induced Mechanical Hyperalgesia: A Functional MRI Study

**DOI:** 10.1155/2013/610727

**Published:** 2013-09-18

**Authors:** Guy H. Hans, Everhard Vandervliet, Kristof Deseure, Paul M. Parizel

**Affiliations:** ^1^Multidisciplinary Pain Center, Antwerp University Hospital and University of Antwerp, Wilrijkstraat 10, 2650 Edegem, Belgium; ^2^Laboratory for Pain Research, University of Antwerp, Wilrijk, Belgium; ^3^Department of Radiology, Antwerp University Hospital and University of Antwerp, Edegem, Belgium

## Abstract

Endothelin-1 (ET-1) is an endogenously expressed potent peptide vasoconstrictor. There is growing evidence that ET-1 plays a role in the pain signaling system and triggers overt nociception in humans. The underlying neuronal pathways are still a matter of great debate. In the present study, we applied an intradermal ET-1 sensitization model to induce mechanical hyperalgesia in healthy subjects. Functional magnetic resonance imaging (fMRI) was used to tease out the cortical regions associated with the processing of ET-1-induced punctate hyperalgesia, as compared to a nonnoxious mechanical stimulation of the contralateral arm. Von Frey hair testing revealed the presence of increased responsiveness to punctate stimulation in all subjects. Activational patterns between nonpainful control stimulation and hyperalgesic stimulation were compared. Two major observations were made: (1) all cortical areas that showed activation during the control stimulation were also present during hyperalgesic stimulation, but in addition, some areas showed bilateral activation only during hyperalgesic stimulation, and (2) some brain areas showed significantly higher signal changes during hyperalgesic stimulation. Our findings suggest that injection of ET-1 leads to a state of punctate hyperalgesia, which in turn causes the activation of multiple brain regions. This indicates that ET-1 activates an extended neuronal pathway.

## 1. Introduction

Pain is a complex and often difficult to treat condition, with different etiologies, locations, and symptoms. The cause of pain is frequently unknown but may involve mediator-dependent signaling from peripheral organs (such as skin or bone) to spinal nerves. Endothelin-1 (ET-1), a 21 amino acid residue peptide, is one such possible pain mediator. Originally recognized as a potent vasoconstrictor [1], ET-1 has been shown to possess nociceptive properties in animals and pain-inducing properties in humans. It has been postulated that ET-1 is involved in driving acute and chronic pain conditions from different etiologies [2].

ET-1 is expressed in neurons of the brain and spinal cord [3–5], as well as in dorsal root ganglia [3, 6]. Likewise, receptors for ET-1 are found in neurons throughout the CNS [5]. Such presence of ET-1 and its receptors in nervous tissues suggests its possible role as a neurotransmitter and/or neuromodulator. Studies have shown that exogenous application of ET-1 produced pain-like behavior in animals [7–11] and pain in humans [2, 12–15]. ET-1 involved in pathological states is released from nonneuronal cells, for example, keratinocytes, cardiomyocytes, and cancer cells [16, 17]. Moreover, endogenous endothelins contribute significantly to the pain and/or hyperalgesia of inflammatory, immune, neuropathic, and neoplastic origins [17–20].

Despite the abundance of scientific evidence documenting the role of ET-1 in pain transmission, little is known about the specific characteristics of this involvement. Animal studies have shown that the injection of ET-1, in addition to causing overt nociception, also induces hyperalgesia to mechanical stimuli [21, 22]. We have performed the first neurosensory evaluation of intradermal injection of ET-1 in humans [23]. In addition to spontaneous pain symptoms, study results indicated the development of long-lasting punctate hyperalgesia. There is emerging evidence that ongoing C-fiber discharge from the peripheral nervous system (PNS) may induce CNS-derived A-fiber-mediated mechanical hyperalgesia. Development and maintenance of this hyperalgesic state require heterosynaptic changes within the CNS, for example, functional changes within the spinal cord or brain [24, 25].

Taking into account our previous experience, in the present study our purpose was to evaluate the cortical activations that occur during an ET-1-induced hyperalgesic state. In order to obtain an insight into the neuronal matrix involved in the central processing of ET-1-induced hyperalgesia, fMRI was applied to identify the cortical regions associated with the processing of this ET-1-induced mechanical hyperalgesia. ET-1-induced brain activation patterns were compared to nonpainful mechanical von Frey probe stimulation.

## 2. Methods

### 2.1. Participants

The study design included nine healthy right-handed volunteers (7 females and 2 males, mean age  29.27 ± 8.62  years). Subject volunteers who responded to advertisements regarding this project were working at the University Hospital. No participant was taking medication or drugs that could interfere with itch or pain sensations and flare response (i.e., analgesics, antihistamines, and calcium or sodium channel blockers). Subjects refrained from alcohol and nicotine use during the 24 hours before the study. Written informed consent was obtained from all participants before the experiments. The study protocol was submitted to and approved by the Ethics Committee of the Antwerp University Hospital and adhered to the tenets of the Declaration of Helsinki.

### 2.2. Experimental Pain Model and Psychophysics

**Figure 1:**
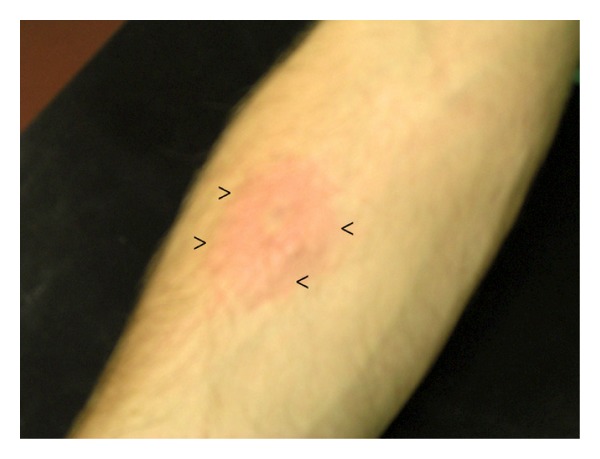
A picture that displays the central pallor zone (adjacent to the injection site), surrounded by a larger area of flare after injection of ET-1. Von Frey filament stimulation sites are indicated by the arrows (<).

Forty-five minutes prior to the scanning session, a single intradermal injection of 40 *μ*L of a 10^−6 ^M ET-1 solution was performed in the volar surface of the a forearm of all participating subjects. Participants were randomized to receive the ET-1 injection in either the right or the left forearm. The injection site was marked with a pen, as was the homologous anatomic region in the contralateral (noninjected) arm. Development of punctate hyperalgesia was tested 10 and 30 min after injection and compared to mechanical stimulation of the contralateral (left) arm. Punctate stimulation was applied using a rigid von Frey monofilament applied at 90 degrees to the skin surface (bending force of 254.9 mN). This von Frey probe, which causes only a sensation of slight discomfort in normal skin, was applied along a line that marked the edge of the visual flare (Figure 1). Subject volunteers were instructed to report the occurrence of a definite change in sensation during this stimulation, often to a more intense stinging with a prolonged aftersensation. The hyperalgesic area was defined as the skin region in which punctate stimulation produced a definite change in the quality of the sensation described by the subjects as “painful,” “burning,” “tenderness,” “more intense pricking,” and “more unpleasant” (from high to low intensity). Subjects were asked to describe the qualitative perception of von Frey hair stimulation in the presence or absence of ET-1 to confirm that the descriptors mentioned above were reported after ET-1 injection only. These response codes have been used previously to monitor development of hyperalgesia in humans [26, 27].

Ten and twenty minutes after intradermal injection of ET-1, subjects were asked to report any sensation of pain. Volunteers rated the intensity of spontaneous pain induced by endothelin-1 using a visual analogue scale (VAS) 10 cm in length and anchored by word descriptors at each end (left-hand end: “no pain” and on the right-hand end: ‘‘the worst imaginable pain”). Subjects marked on the line at the point that they felt represented with their current state of nociception.

**Figure 2:**
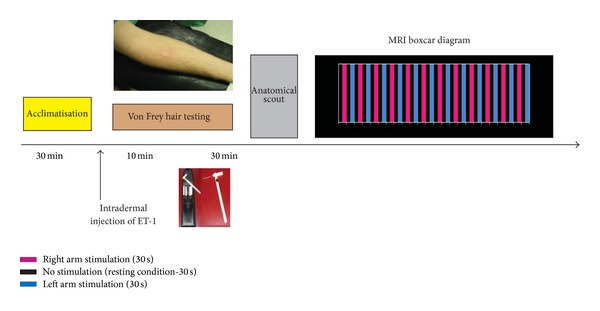
Schematic illustration of the experimental protocol. Following intradermal injection of ET-1 on the forearm, responses to mechanical stimulation were tested after 10 and 30 min. Following acquisition of the anatomical scout, a block design was employed with two conditions (stimulus and baseline). Each stimulus block consisted of 12 blocks of tactile stimulation of the injection site alternated by as many blocks of contralateral (noninjected) arm stimulation. Both conditions were separated by a resting condition that consisted of no stimulation whatsoever. Each of the stimulation blocks lasted for 30 seconds.

A detailed overview of the experimental design used for our fMRI experiments is provided in Figure 2. The paradigm used was of the boxcar type consisting of 12 blocks of tactile stimulation of the injection site alternated by as many blocks of contralateral left arm stimulation. Both conditions were separated by a resting condition, which consisted of no stimulation whatsoever. Each of the blocks lasted for 30 seconds, amounting in 24 minutes of functional scanning ([*rest* - *right* - *rest* - *left*] × 12). During the fMRI scans, hyperalgesia was rekindled by continuous tactile stimulation (every two seconds one stimulation was performed, controlled by the use of a stopwatch) performed by one of the authors (GH), using the same rigid monofilament as mentioned before. Mechanical stimulation was always first performed on the control, noninjected side followed by mechanical stimulation of the injected (hyperalgesic) side.

### 2.3. fMRI and Acquisition

#### 2.3.1. Image Acquisition

Functional MR images were collected on a 1.5-Tesla superconducting magnet (Magnetom Sonata, Siemens, Erlangen, Germany) equipped with 40 mT/m gradients and a standard circularly polarized head coil, using a BOLD sensitive T2-weighted single shot gradient-recalled echo (GRE) echo planar imaging (EPI) sequence (TE/TR 50/3000 ms) resulting in voxel dimensions of 3 × 3 × 3 mm^3^. In this way, we acquired 240 volumes consisting of 30 slices each, both during baseline and under the conditions of interest. In the same scanning session, we also recorded a T1-weighted magnetization prepared rapid acquisition gradient recalled echo series (MP-RAGE; 1 × 1 × 1 mm^3^; TE/TR 3.76/1700 ms) and a T1-weighted spin echo series (SE; 1 × 1 × 1.5 mm^3^; TE/TR 15/700).

### 2.4. fMRI Data Analysis

For all data processing and analysis, we used a commercially available and dedicated software package (Brain Voyager QX software package, version 1.3.8; Brain Innovation, Maastricht, The Netherlands). Based on DICOM header information, EPI images were linked to the SE T1-weighted anatomical images and the resulting volume was then fitted into the three-dimensional MP-RAGE anatomical dataset. Preprocessing included 4 mm Gaussian spatial smoothing (FWHM), high pass filtering and linear trend removal, three-dimensional motion correction, and slice scan time correction. Afterwards, the individual data were transformed into the standard stereotactic space as described by Rey et al. [28]. For both the individual and the group analysis, the condition of interest was convoluted with a hemodynamic response function, as introduced by Boynton, and served as an independent predictor in the general linear model (GLM) [29]. Voxels were considered to be activated when their time courses followed the model used in the GLM, and voxel activity was considered to be significant above the *t*-value that coincided with a false discovery rate of 5 percent. To further minimize false positive voxels, the minimal threshold for contiguous clusters to be depicted in the statistical map was set to 100. 

### 2.5. Statistical Analysis

The mean maximum signal changes of all brain areas encountered during hyperalgesic stimulation were compared to those of corresponding areas on the contralateral noninjected side. BrainVoyager QX was used for all statistical analyses. To assess statistically significant differences between the areas of hyperalgesia, a student's *t*-test for matched pairs was employed. *P* < 0.05 was considered statistically significant. Response scores to punctate stimulation were evaluated using nonparametric analysis (Friedman test), with significance levels of *P* < 0.05. To compare the data of spontaneous pain measurement, two-way repeated measurement ANOVA (two-way RM ANOVA) was performed, and a post-hoc Student-Newman-Keul's for pairwise multiple comparison was made if the ANOVA was significant. Nonparametric and ANOVA analyses were performed using Prism's statistical software (version 6.0b for Mac).

## 3. Results

### 3.1. Psychophysical Test Session

**Figure 3:**
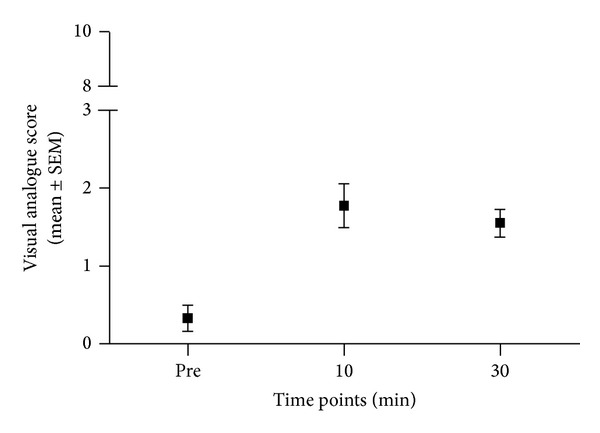
Graph showing the time course of visual analogue scores (VAS) after intradermal injection of endothelin-1 (ET-1). No significant alterations were observed *over time* (*P* > 0.05).

**Figure 4:**
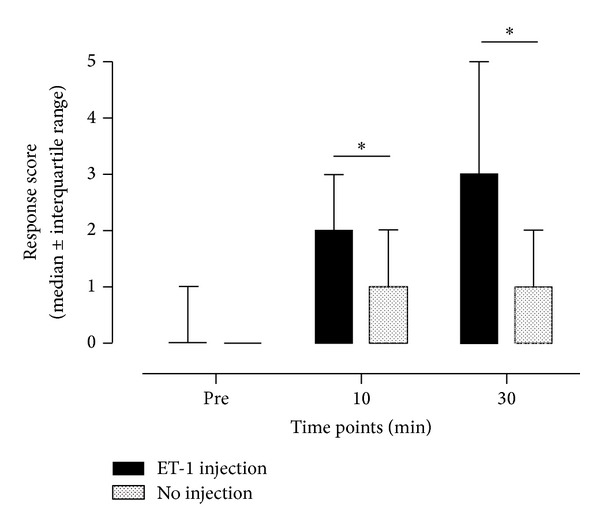
Time course of changes in response code to punctate stimulation. The following response codes were applied: normal sensation (as indicated by 0 in the *Y*-axis of the graph); more unpleasant (1), more intense pricking (2), tenderness (3), burning (4), and painful (5). Data are expressed as median values ± interquartile range. Injection of 10^−6 ^M ET-1 induced a significant increase in response code compared to no injection, as indicated by the asterisks (Friedman test, *P* < 0.05). Response codes after ET-1 injection were significantly increased after both 10 and 30 min.

None of our test subjects reported any significant spontaneous pain sensations on the ET-1 injected arm (Figure 3). In contrast, ten minutes after the ET-1 injection, subjects started to report a hyperalgesic state to von Frey filament stimulation (Figure 4). Compared to mechanical stimulation of the noninjected arm, subjects displayed a significant increase in responsiveness to punctate stimulation at the lateral border of the flare area (*P* < 0.05). The intensity of punctate hyperalgesia increased even more 30 min after the injection of ET-1.

### 3.2. Cortical Activation during Mechanical Stimulation on the Unaffected Side

Mechanical stimulation of the contralateral, noninjected arm resulted in significant activations of different brain regions (Table 1). Ipsilateral activation was observed in Brodmann areas (BA) 2, 4, 5, and 6 as well as in supplementary motor area (SMA) 8, 9, 22, 30, 47, and the amygdala. Bilateral activation occurred in BA 6 (premotor cortex, M2), 13, 18, 19, 37, 44, and 46.

### 3.3. Cortical Activation in Response to the Provocation of Mechanical Hyperalgesia

**Figure 5:**
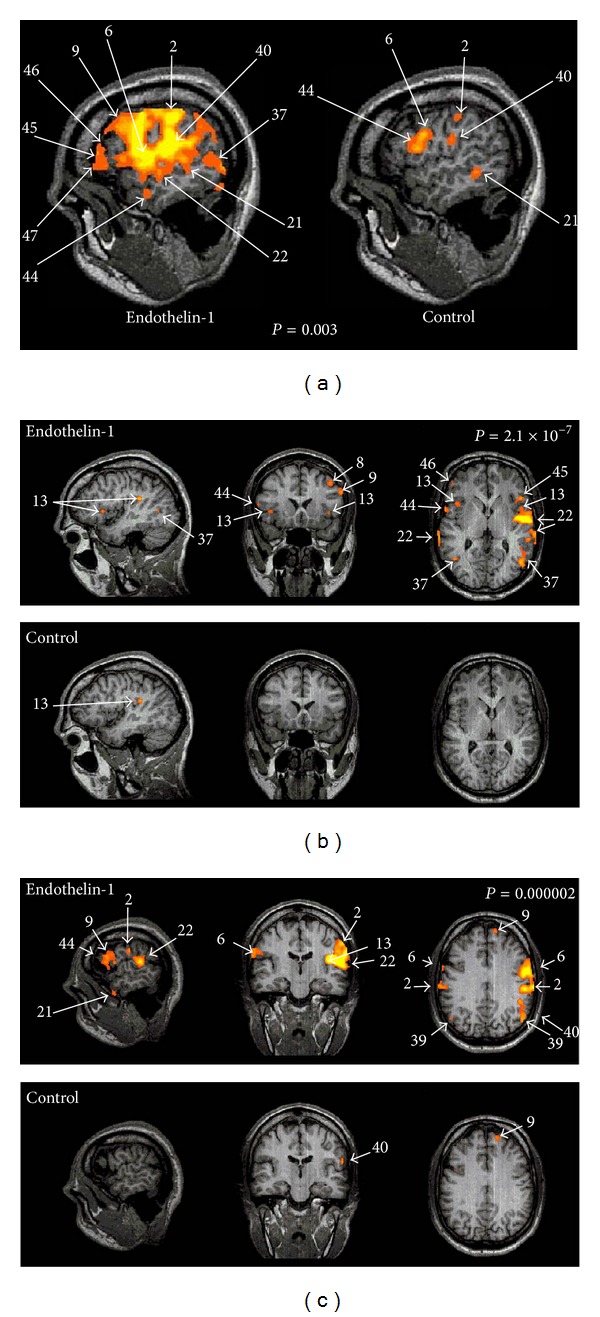
Cerebral activation in response to ET-1-induced provocation of punctate hyperalgesia at the level of the Anterior Cingulate Cortex (a), at the level of the Insula (b), and the Somatosensory Cortex (c). The results of von Frey filament stimulation of the hyperalgesic right arm contrasted with the results from identical stimulations of the left (noninjected) arm.

Several cortical and subcortical brain areas exhibited significant activation in response to the provocation of punctate hyperalgesic pain (Table 2). All areas observed during control stimulation were also activated during hyperalgesic stimulation. However, some areas that showed unilateral activation during control stimulation displayed bilateral activation during hyperalgesic stimulation (Table 3(a)). Brain areas with bilateral activation after ET-1 injection included the postcentral gyrus (BA 2, component of the primary somatosensory cortex), SMA/premotor cortex, inferior temporal gyrus (BA 22), and the associational cortical area located in the angular gyrus (BA 39). In addition, several brain areas showed significantly higher mean maximum signal changes only during hyperalgesic stimulation and not during nonpainful stimulation (Table 3(b)). The brain areas exhibiting significant larger signal changes during ET-1 stimulation consist of the prefrontal cortex, anterior insular cortex, occipital lobe (medial and lateral aspects), posterior temporal lobe, and dorsolateral prefrontal cortex. Finally, significant increases in signal changes were also observed in the inferior frontal gyrus (IFC), the anterior cingulate cortex (ACC), and the amygdala (Figure 5).

## 4. Discussion

The purpose of this study was to examine the neuronal matrix that is activated during cerebral processing of endothelin-1-(ET-1) induced hyperalgesic states. Lately, there has been a renewed interest in the pronociceptive effects of ET-1, since the peptide is being increasingly implicated as a mediator in cancer pain [2, 30–33] as well as neuropathic pain [34]. It is now a well-established fact that ET-1 has both pain-producing as well as pain-potentiating properties [7, 18, 20, 21, 35, 36], thereby, both stimulate nociceptors as well as sensitizing them to painful stimuli. In addition to spontaneous nociceptive symptoms, studies in animals have shown that, following cutaneous application of ET-1, secondary punctate hyperalgesia may develop in the affected region of the skin [22, 37]. Recently, we were able to show the development of a long-lasting secondary hyperalgesia to punctate stimuli in human volunteers after intradermal injection of ET-1 [23]. Secondary hyperalgesia likely results from the sensitization of nociceptive neurons in the central nervous system [38–41]. Based on these assumptions, the challenge is therefore to identify the brain areas involved in the processing of this somatosensory state using fMRI. Afterwards, these ET-1-elicited activation patterns should be compared to non-endothelin-induced nociceptive states.

Nociceptive information is transmitted from the spinal cord to the brain via several different pathways. Consequently, multiple regions of the brain are activated during painful experiences. Although there are many differences in activation patterns across studies, brain imaging in humans has demonstrated a consistent cortical pain network the consisting of the primary (S1) and secondary somatosensory cortices (S2), insular cortex (IC), prefrontal cortex (PFC), and the anterior cingulate (ACC) cortices [42]. Although there have now been dozens of human brain imaging studies, most previous fMRI studies were undertaken during experimental nociceptive pain [43–46], whereas ET-1-induced pain should be considered as neuropathic in origin. Only more recently, fMRI studies have begun investigating the neural correlates of neuropathic pain, hereby, mostly focusing on the cortical activations associated with allodynia and hyperalgesia [47, 48]. A previous imaging study of experimental allodynia demonstrated activation in a cortical network comprising S1, S2, PA, IFC, and insular cortices [47]. Other studies showed lesser extent of brain activation patterns in conditions of experimental allodynia [49, 50]. 

### 4.1. Coding of ET-1-Evoked Nociception

Our results show that ET-1-induced punctate hyperalgesia recruits a complex brain network, that involves all areas also found during nonpainful tactile stimulation. During hyperalgesic stimulation, the activated brain areas are not only more active (higher fMRI signal), but the network also becomes more complex with more areas involved in the activation process. The most active cortical areas identified within this network were the primary (S1) and secondary somatosensory cortices (S2), the insula, inferior parietal lobe (IPL), superior frontal cortex (SFC), inferior frontal cortex (IFC), and anterior cingulate cortex (ACC). 

The activation of the primary somatosensory cortex observed in the present study is in line with several reports investigating experimental or clinical forms of allodynia [47, 48, 51–54]. Nociceptive-specific neurons in S1 are sparse and intermingled with neurons of other sensory modalities. The S1 cortex appears to be involved in the sensory-discriminative aspect of pain through a solid link with the ventrobasal region of the thalamus. The observed S1 activations during control stimulation are also in agreement with previous functional imaging studies [47]. 

The insula has also been reliably activated in human pain imaging [47]. The insular cortex is known as a central station of pain processing, and it plays a role in various aspects of pain perception (e.g., affective components of acute pain) [55]. In our study, we observed a significant activation of the anterior insular cortex (rostral part), which matches the portion of the insula where encoding of perceived intensity of experimental pain in healthy volunteers has been consistently described in some previous studies [56]. The insula showed no activation in response to nonpainful von Frey filament stimulation, which is also in accordance with previous findings [57]. 

In addition to the insula, the ACC has also often been implicated in the perception of pain. The mid-ACC has been suggested to be important for the integration of basic nociceptive information with pain perception [58]. The slightly lower intensity (lower  *t*_max⁡_) of ACC activation observed in our study could be linked to the low intensity of ongoing (spontaneous) pain sensed by our volunteers. A previous study investigating neural correlates of ongoing pain intensity showed indeed that ACC activation encodes perceived ongoing pain intensity [59]. The observed activation of ACC in this study, together with activation of SMA, could represent the selection of motor responses to the hyperalgesic, stimulations [60], to facilitate quick flight responses [58].

During the presence of mechanical hyperalgesia we noticed strong activation in the dorsolateral prefrontal cortex (PFC). There is an emerging evidence that BA 9 and 46 are involved in the mapping of extra personal space and surrounding, maintenance of short-term memory, and planning of adequate responses to external stimuli [61]. Furthermore, these brain regions have also been implied previously in pain-related attention processing [62, 63]. The observed prefrontal activity under our experimental pain condition could therefore be interpreted as a consequence of attention, a cognitive evaluation, and a planning of motor behavior in response to ET-1-induced nociception. Finally we observed significant activation patterns in the inferior frontal cortex (IFC). This finding seems to be in agreement with previous functional magnetic resonance imaging studies during experimental allodynia [47]. This brain region seems to be part of the brain processes underlying stimulus-evoked pain. 

An interesting finding lies in the strong activation of the amygdala in the contralateral hemisphere which was observed during von Frey filament stimulation of the ET-1 injected arm. The amygdala belong to the emotional circuitry of the brain, which is only rarely activated in acute pain conditions. Therefore, little is known regarding the role of the amygdala in nociceptive conditions. Activation of these brain areas in our experimental setting is therefore challenging and could be linked to the emotional meaning of the ET-1-induced hyperalgesic condition. Previously, it was discovered that the amygdala receives direct nociceptive projections from nonpeptidergic IB4 neurons [64]. In contrast, the spinothalamic pathways are mainly composed of CGRP peptidergic afferents and are thought to mediate nociceptive inputs to S1, S2, and parts of insula. It therefore seems that intradermal injection of ET-1 leads to a pronounced hyperalgesic state, inducing activation of multiple neuronal pain-related circuitries. The evoked nociception seems to recruit both sensory-representational cortical areas as well as hedonic subcortical areas.

Another challenging finding of our study was the absence of any specific activation of the PA cortex during ET-1-induced hyperalgesia. This is possibly due to the fact that only a very low dose of ET-1 was injected, resulting in a rather limited area of moderate hyperalgesia with low levels of spontaneous pain. In fact, results from previous studies have indicated that activation of the PA cortex could be highly dependent on the intensity of the painful stimulus [47].

The findings of this study open a couple of interesting perspectives. First of all, the brain activation pattern induced by von Frey filament stimulation of ET-1 injected skin seems to largely correspond with the activation patterns observed after mechanical stimulation of capsaicin treated skin [47, 65]. The observed activation pattern is distinct from the one during thermal hyperalgesia [65]. This can be considered a further proof that mechanical (secondary) hyperalgesia not only has its distinct coding in the human brain, but also that the same neurological symptom is coupled with to the same gross neuronal network regardless of the underlying etiology (capsaicin or ET-1) that causes the development of a secondary hyperalgesic syndrome. Moreover, the finding seems to indicate that ET-1 activates a rather “classical” sensory pathway to induce nociception. However, some differences still exist with other experimental pain conditions. ET-1 seems to activate brain regions that had not been identified in earlier functional imaging studies, whereas other brain regions show no activation during ET-1-induced hyperalgesia. These differences are probably linked to different psychophysical properties and warrant further investigations.

Finally, it should be stressed that the currently described fMRI findings relate to an acute (single) intradermal injection of ET-1 in healthy volunteers. It is quite conceivable that these fMRI findings would be somewhat different obtained from patients showing long-term sensitization induced by continuous (daily) elevation of endogenous ET-1 levels, such as in cancer conditions or other disease states. One should be aware of this restriction when interpreting the results of this study. Therefore, in a subsequent study, we are planning to apply the same fMRI protocol in patients suffering from malignant pain induced by cancer types which are known to induce significantly elevated levels of ET-1 (such as prostate cancer or melanoma).

## 5. Conclusion

We have demonstrated that our ET-1-induced hyperalgesic model induces activation of a complex neuronal network. Considering the growing interest of ET-1 in cancer pain as well as other neuropathic pain conditions, this new human experimental mode of tonic application of ET-1 could prove to be essential to the further unraveling of endothelin-induced nociception. Additional studies are therefore warranted to further explore the specific features of the ET-1 activated neuronal matrix. The fact that the subject volunteers displayed minimal spontaneous pain sensations after intradermal injection of ET-1 could prove to be of great practical value for future functional imaging studies. Indeed, most experimental models of allodynia and hyperalgesia suffer from methodological limitations due to the continuous presence of intense (burning) pain, which compromises the dissociation between evoked and spontaneous nociceptive symptoms.

## Figures and Tables

**Table 1:** Regions of cerebral activations during nonpainful stimulation of the noninjected arm.

Lobus	Gyrus	Side	Talairach			BA	*t*_max⁡_	Lowest *P* value
			*x *	*y *	*z *			
Innocuous mechanical stimulation								
Frontal lobe	Pre-central	Ipsilateral	−42	−10	55	4	3.4	0.0051
Frontal lobe		Contralateral	21	−42	56	5	6.4	0.0000000228
Frontal lobe		Contralateral	1	−6	60	6	4.4	0.0001
Frontal lobe		Contralateral	51	1	30	6	4.2	0.00034
Frontal lobe		Ipsilateral	−54	1	24	6	7.4	2.78*e* − 09
Frontal lobe		Ipsilateral	−13	52	31	9	6.4	9.78*e* − 09
Frontal lobe		Contralateral	40	42	8	46	4.8	0.000002
Frontal lobe	Middle frontal	Ipsilateral	−41	44	5	46	6	0.000001
Frontal lobe	Middle frontal	Ipsilateral	−43	38	1	47	5.2	0.000002
Frontal lobe		Contralateral	52	5	14	44	5.6	0.00000021
Frontal lobe	Inferior frontal	Ipsilateral	−53	5	14	44	6.9	0.000000022
Parietal lobe		Contralateral	42	−59	40	40	4	0.0014
Parietal lobe		Ipsilateral	−55	−26	37	2	4.8	0.00032
Parietal lobe		Ipsilateral	−36	−62	35	39	5	0.000011
Parietal lobe		Ipsilateral	−57	−20	20	40	6.8	0.0000000021
Temporal lobe		Ipsilateral	−15	36	42	8	5.5	0.000004
Temporal lobe		Contralateral	41	−61	4	37	5.8	0.000002
Temporal lobe		Contralateral	53	0	5	22	5.6	0.00024
Occipital lobe		Contralateral	29	−83	12	19	4.9	0.000011
Occipital lobe		Contralateral	24	−90	−5	18	5.7	0.00000021
Occipital lobe		Ipsilateral	−41	−67	−1	37	4	0.0027
Occipital lobe	Middle occipital	Ipsilateral	−26	−85	14	19	5.4	0.000002
Occipital lobe	Middle occipital	Ipsilateral	−28	−87	2	18	6.6	0.000000022
Limbic lobe		Contralateral	14	−39	−6	30	5	0.000011
Insula		Contralateral	43	−34	22	13	6.9	1.2*e* − 11
Insula	Sublobar	Ipsilateral	−49	−38	20	13	3.6	0.001
Amygdala		Ipsilateral	−22	−7	−10		3.6	0.0023
Limbic lobe		Contralateral	6	−6	45	24	4.9	1.1*e* − 05
Limbic lobe		Ipsilateral	−10	−7	42	24	3.2	1.2*e* − 03

**Table 2:** Regions of cerebral activations during painful stimulation of the ET-1 injected arm.

Lobus	Gyrus	Side	Talairach			BA	*t*_max⁡_	lowest *P* value
			*x *	*y *	*z *			
Noxious mechanical stimulation								
Frontal lobe		Ipsilateral	60	3	24	6	5.9	1.51*e* − 08
Frontal lobe		Contralateral	−2	−4	60	6	4.2	0.00005
Frontal lobe		Contralateral	−50	−3	41	6	8.9	7.07*e* − 13
Frontal lobe		Contralateral	−16	46	36	8	5.8	2.69*e* − 07
Frontal lobe		Contralateral	−11	56	30	9	6.8	0.000000209
Frontal lobe		Ipsilateral	50	41	8	46	5.6	0.000002
Frontal lobe		Contralateral	−41	34	16	46	6.2	0.0000000228
Frontal lobe		Contralateral	−44	22	2	47	7	2.14*e* − 09
Frontal lobe		Ipsilateral	58	9	13	44	6.4	0.0000000228
Frontal lobe		Contralateral	−51	1	19	44	11.7	2.73*e* − 023
Frontal lobe		Ipsilateral	9	52	30	9	3.9	0.00068
Frontal lobe		Ipsilateral	5	16	52	8	2.9	0.0039
Parietal lobe		Contralateral	−54	−25	38	2	9.6	5.45*e* − 020
Parietal lobe		Ipsilateral	50	−43	43	40	4.3	0.000064
Parietal lobe		Contralateral	−45	−26	20	40	13.5	1.17*e* − 32
Parietal lobe		Ipsilateral	49	−61	32	39	5.4	0.000001
Parietal lobe		Ipsilateral	55	−21	30	2	6.1	0.00000015
Temporal lobe		Contralateral	−65	−27	5	22	7.3	1.72*e* − 10
Temporal lobe		Ipsilateral	45	−61	4	37	6.8	2.09*e* − 07
Temporal lobe		Contralateral	−46	−65	3	37	7.8	1.19*e* − 11
Temporal lobe		Contralateral	−49	−62	24	39	7.9	2.14*e* − 09
Temporal lobe		Ipsilateral	67	−32	6	22	7.1	1.72*e* − 010
Occipital lobe		Ipsilateral	34	−89	9	19	2.9	0.0094
Occipital lobe		Contralateral	−35	−89	7	19	3.5	0.003
Occipital lobe		Contralateral	−27	−85	−7	18	3.9	0.00034
Occipital lobe		Ipsilateral	33	−89	1	18	3.6	0.00035
Amygdala		Contralateral	−27	−2	−10		5.1	0.000005
Limbic lobe		Ipsilateral	16	−39	−8	30	3.4	0.0093
Limbic lobe		Ipsilateral	5	−6	45	24	4.1	0.000082
Limbic lobe		Contralateral	−12	−3	47	24	3.1	0.0011
Insula		Ipsilateral	44	−35	21	13	8	5.99*e* − 7
Insula		Contralateral	−46	−26	20	13	13.3	3.4*e* − 31

**Table:** (a) Bilateral activation after ET-1 versus unilateral activation after innocuous stimulation

Lobus	BA	Lowest *P* value
Parietal lobe	2	0.005
Frontal lobe	8	0.007
Frontal lobe	9	0.0013
Temporal lobe	22	0.002
Temporal lobe	39	0.023

**Table:** (b) Unilateral activation after ET-1 versus no activation after innocuous stimulation

Lobus	BA	Lowest *P* value
Parietal lobe	2	0.0004
Frontal lobe (PMC)	6	0.02
Frontal lobe	9	0.008
Insula	13	0.004
Occipital lobe	18	0.01
Temporal lobe	37	0.01
Frontal lobe	44	0.04
Frontal lobe	46	0.0005
Frontal lobe	47	0.0004
Amygdala		0.03
